# Diagnostic Potential of the Plasma Lipidome in Infectious Disease: Application to Acute SARS-CoV-2 Infection

**DOI:** 10.3390/metabo11070467

**Published:** 2021-07-20

**Authors:** Nicola Gray, Nathan G. Lawler, Annie Xu Zeng, Monique Ryan, Sze How Bong, Berin A. Boughton, Maider Bizkarguenaga, Chiara Bruzzone, Nieves Embade, Julien Wist, Elaine Holmes, Oscar Millet, Jeremy K. Nicholson, Luke Whiley

**Affiliations:** 1 Australian National Phenome Centre, Health Futures Institute, Harry Perkins Institute, Murdoch University, 5 Robin Warren Drive, Perth, WA 6150, Australia; nicola.gray@murdoch.edu.au (N.G.); nathan.lawler@murdoch.edu.au (N.G.L.); annie.zeng@murdoch.edu.au (A.X.Z.); monique.ryan@murdoch.edu.au (M.R.); s.bong@murdoch.edu.au (S.H.B.); berin.boughton@murdoch.edu.au (B.A.B.); julien.wist@murdoch.edu.au (J.W.); elaine.holmes@murdoch.edu.au (E.H.); 2 Centre for Computational and Systems Medicine, Health Futures Institute, Harry Perkins Institute, Murdoch University, 5 Robin Warren Drive, Perth, WA 6150, Australia; 3 Centro de Investigación Cooperativa en Biociencias—CIC bioGUNE, Precision Medicine and Metabolism Laboratory, Basque Research and Technology Alliance, Bizkaia Science and Technology Park, Building 800, 48160 Derio, Spain; mbizcarguenaga@cicbiogune.es (M.B.); cbruzzone@cicbiogune.es (C.B.); nembade@cicbiogune.es (N.E.); 4 Chemistry Department, Universidad del Valle, Cali 76001, Colombia; 5 Department of Metabolism Digestion and Reproduction, Faculty of Medicine, Imperial College London, Sir Alexander Fleming Building, South Kensington, London SW7 2AZ, UK; 6 Institute of Global Health Innovation, Faculty Building South Kensington Campus, Imperial College London, London SW7 2AZ, UK; 7 Perron Institute for Neurological and Translational Science, Nedlands, WA 6009, Australia

**Keywords:** metabolic phenotyping, infectious disease, lipids, lipidomics, liquid chromatography-mass spectrometry (LC-MS), SARS-CoV-2, COVID-19

## Abstract

Improved methods are required for investigating the systemic metabolic effects of SARS-CoV-2 infection and patient stratification for precision treatment. We aimed to develop an effective method using lipid profiles for discriminating between SARS-CoV-2 infection, healthy controls, and non-SARS-CoV-2 respiratory infections. Targeted liquid chromatography–mass spectrometry lipid profiling was performed on discovery (20 SARS-CoV-2-positive; 37 healthy controls; 22 COVID-19 symptoms but SARS-CoV-2negative) and validation (312 SARS-CoV-2-positive; 100 healthy controls) cohorts. Orthogonal projection to latent structure-discriminant analysis (OPLS-DA) and Kruskal–Wallis tests were applied to establish discriminant lipids, significance, and effect size, followed by logistic regression to evaluate classification performance. OPLS-DA reported separation of SARS-CoV-2 infection from healthy controls in the discovery cohort, with an area under the curve (AUC) of 1.000. A refined panel of discriminant features consisted of six lipids from different subclasses (PE, PC, LPC, HCER, CER, and DCER). Logistic regression in the discovery cohort returned a training ROC AUC of 1.000 (sensitivity = 1.000, specificity = 1.000) and a test ROC AUC of 1.000. The validation cohort produced a training ROC AUC of 0.977 (sensitivity = 0.855, specificity = 0.948) and a test ROC AUC of 0.978 (sensitivity = 0.948, specificity = 0.922). The lipid panel was also able to differentiate SARS-CoV-2-positive individuals from SARS-CoV-2-negative individuals with COVID-19-like symptoms (specificity = 0.818). Lipid profiling and multivariate modelling revealed a signature offering mechanistic insights into SARS-CoV-2, with strong predictive power, and the potential to facilitate effective diagnosis and clinical management.

## 1. Introduction

The COVID-19 pandemic, caused by severe acute respiratory syndrome coronavirus 2 (SARS-CoV-2), remains a threat to public health across the world, with emerging variants and growing evidence of long-term health concerns post-infection. Despite a global response from the scientific community to enhance our understanding of viral pathogenesis, efforts are still ongoing to enhance acute diagnosis and support patient stratification. Currently, the gold standard for the diagnosis of SARS-CoV-2 infection is reverse transcription PCR (RT-PCR). However, its effectiveness has been questioned throughout the pandemic, with a false negative rate reported to be up to 20%, dependent on factors including sampling time post-infection, low virus titre, sampling error, and experimental error [1]. Furthermore, RT-PCR testing only has a small sampling time window, and gives no indication of subsequent clinical outcome—for example, the risk of severe disease, or post-acute COVID-19 syndrome (PACS). Therefore, there is an urgent need for new tools that can better stratify patients and help to augment existing RT-PCR strategies and mitigate the diagnostic limitations in the clinical management of the disease.

The concept of metabolomic phenotyping and lipidomic approaches as diagnostic tools using ultra-high-pressure liquid chromatography coupled with mass spectrometry (UHPLC–MS) technologies is well established in the field of inborn errors of metabolism [2,3,4], and has more recently been demonstrated in large-scale biomedical research to uncover biomarkers associated with infectious disease and discriminate specific infectious pathogens [5]. With respect to SARS-CoV-2, early studies have indicated that infection results in changes in an individual’s phenotype, leading to phenoconversion (the change from a normal or healthy state to a disordered pathophysiological state or overt pathology [6]), which is reflected in perturbations in specific metabolic pathways that are associated with infection [6,7,8,9,10].

Recent reports have detailed changes in plasma lipoproteins and the lipidome in response to SARS-CoV-2 infection [11,12]. Lipids are critical to systems biology, with essential roles as membrane structural components, signalling molecules, and energy sources, meaning that the lipidome is a rich source of biological information that has great potential in diagnostic pathology. Furthermore, due to the vast range of functions that lipids have in the human system, profiling the human plasma lipidome can provide mechanistic insights into the pathogenesis of infection. Therefore, lipidomic characterization of patients with infection has the potential to not only identify new diagnostic lipid biomarkers but also to improve the mechanistic understanding of infection, and facilitate long-term monitoring and impact on health post-infection.

Lipid perturbations have previously been observed in critical illness and viral infection, with viruses known to induce profound changes in host cell lipidomes and key energy pathways in their exploitation of host cell metabolic resources to fuel the different stages of viral infection [13]. Dramatic alterations in the human plasma lipidome have been noted following viral infections such as Ebola virus disease [14,15], human immunodeficiency virus (HIV) [16], hepatitis B virus [16], hepatitis C virus [17], Zika virus [18], and influenza [19], as well as severe acute respiratory syndrome coronavirus (SARS-CoV) [20] and Middle East respiratory syndrome (MERS) [21]. In addition to alterations noted during acute infection, significant lipidomic changes have been observed in SARS patients 12 years following infection [20]. Furthermore, extensive lipid metabolism remodelling has been reported in cells infected with human coronaviruses, including alterations in free fatty acids and glycerophospholipids [21].

In SARS-CoV-2 infection, multiple plasma lipids have been reported to alter in response to the disease, suggesting that the lipidome could be a potential source of biomarkers with diagnostic capability. For example, higher concentrations of plasma triglycerides (TAGs) and lower concentrations of cholesterol esters (CE) were reported in patients with severe COVID-19 symptoms compared to those with mild symptoms [22], whilst similar trends in higher circulating TAGs have been reported elsewhere [12,23,24]. The potential for diagnostic markers that can better stratify patient response to infection is highlighted in reports that indicated higher levels of diglycerides (DAGs), free fatty acids (FFAs), and triglycerides (TAGs) in fatal cases of COVID-19, and that abundance was correlated with deterioration of the disease [25]. However, the requirement for the validation of lipid biomarkers in multiple independent cohorts is highlighted by conflicted reports on phospholipid changes, with increases in phosphocholines (PCs) and phosphoethanolamines (PEs) reported [12,26], as well as significant downregulation [23]. Furthermore, Song et al. identified decreases in longer chain TAGs and DAGs [27], in contrast to the previously discussed studies.

Since metabolite and lipid profiling have emerged as promising tools for the identification of novel diagnostic and prognostic markers of infectious disease, we sought to characterize the plasma and serum lipidome associated with SARS-CoV-2 infection. We performed comprehensive plasma and serum lipidomic analyses using targeted liquid chromatography–tandem mass spectrometry (LC–MS/MS) to examine 845 lipid species originating from 19 lipid classes. Critically, two independent cohorts were used for the discovery and validation of potential biomarkers. The initial discovery cohort consisted of 79 samples collected in Australia (20 SARS-CoV-2-positive individuals, 37 healthy controls, and 22 SARS-CoV-2-negative individuals reporting COVID-19 symptoms but PCR negative for SARS-CoV-2), and the validation cohort consisted of 412 samples collected in Spain (312 SARS-CoV-2-positive; 100 healthy controls).

Quantitative targeted lipidomic analyses revealed a strong lipidomic signature that could distinguish between the SARS-CoV-2-positive and healthy control groups in both cohorts. A panel of six lipids is proposed with excellent diagnostic capability, with the additional benefit of providing mechanistic information on disease pathogenesis, further improving our understanding of the systemic response to infection.

## 2. Methods

### 2.1. Discovery Cohort Patients and Sample Collection (Australia)

The discovery cohort consisted of samples collected from Perth, Australia. The study was initiated at Fiona Stanley Hospital by the COVID Research Response Collaboration (https://research–au.net/covid–research–response/, accessed on 9 June 2021) as part of the International Severe Acute Respiratory and Emerging Infection Consortium (ISARIC)/World Health Organisation (WHO) pandemic trial framework (SMHS Research Governance Office PRN:3976 and Murdoch University Ethics no. 2020/052, and no. 2020/053). A description of the cohort, including demographic data, is provided in Table 1.

Plasma samples were collected from 79 individuals who were either (1) SARS-CoV-2 positive (*n* = 20) individuals; (2) healthy control individuals who were recruited at a similar time of SARS-CoV-2 (+) patient sample collection, but displayed no signs or symptoms of infection (*n* = 37); or (3) SARS-CoV-2-negative (*n* = 22) individuals who reported signs and symptoms of COVID-19 disease but tested negative for SARS-CoV-2 infection.

### 2.2. Validation Cohort Patients and Sample Collection (Spain)

A cohort of serum samples were obtained from collaborators at the Centro de Investigación Cooperativa en Biociencias—CIC bioGUNE, Derio, Bizkaia, Spain. Patients were recruited in the Basurto University Hospital and Cruces University Hospital in the Basque Country (Spain), with 312 positive SARS-CoV-2 patient samples and 100 healthy control samples used for study validation. The project was evaluated and approved by the Comité de Ética de Investigación con Medicamentos de Euskadi (CEIm-E, PI + CES-BIOEF 2020-04 and PI219130). All samples were supplied by the Basque Biobank for Research (BIOEF). Samples were imported under Import Permit 0004275122 issued by the Australian Government’s Department of Agriculture, Water, and the Environment.

### 2.3. LC–MS/MS Lipid Analysis

Lipid analysis was performed by ultra-high-performance liquid chromatography–tandem mass spectrometry (UHPLC–MS/MS) using a targeted approach using predefined MRM transitions (Sciex sMRM Pro Builder, Framingham, MA, USA) and in-house chromatographic retention time windows. Plasma and serum samples (10 μL) were placed in a 96-well plate, to which 90 μL extraction solvent (propanol-2-ol) containing stable-isotope-labelled internal standards (Lipidyzer^TM^ Internal Standards Kit from Sciex (Framingham, MA, USA), SPLASH LipidoMIX^TM^, Lyso PI 17:1, Lyso PG 17:1, and Lyso PS 17:1 (Avanti Polar Lipids, Alabaster, AL, USA)) was added. Samples were shaken for 10 min and stored at −20 °C for 20 min before being centrifuged at 3900× *g* for 15 min. Supernatant was then transferred to a 96-well plate for analysis. For quality control (QC), an independent pooled sample underwent repeat extractions and was injected onto the system at frequent intervals throughout the run. The analytical platform is described in detail in the Supplementary Materials.

### 2.4. Data Pre-Processing and Quality Control

Data were pre-processed using SkylineMS (Seattle, WA, USA) [28], and underwent additional pre-processing and quality control (QC) procedures using in-house pipelines developed in R. This included feature filtering, %RSD < 30% in the replicate QC extracts, and signal intensity threshold filtering. Signal drift was accounted for using random forest signal correction (QC-RFSC) from the statTarget package in R (https://stattarget.github.io, accessed on 9 June 2021).

### 2.5. Statistics

All statistics and data visualisations were generated in R (v.4.0.3) and R Studio (v. 1.3.959) (Boston, MA, USA).

For univariate comparisons, Mann–Whitney rank sum tests were performed on each lipid and compared between the two groups: SARS-CoV-2-positive individuals versus healthy controls. To control the false discovery rate (FDR), *q*-values [29] were generated from the Kruskal–Wallis *p*-values, using the method proposed by Benjamini and Hochberg [30]. The Cliff’s delta statistic was calculated for the 845 measured lipids in order to assess the overall effect size for the intergroup differences. Absolute Cliff’s delta scores were interpreted as 1 indicating maximum difference and 0 indicating no difference.

Supervised multivariate statistical modelling was performed with the combined set of 845 analytes. Data were log-transformed and autoscaled prior to modelling. Multivariate orthogonal projection to latent structures discriminant analysis (OPLS-DA) was performed using the R package metabom8 (version 0.2), available from GitHub (github.com/tkimhofer/metabom8, accessed 9 June 2021).

Logistic regression was performed using the glm method in the R package caret (https://CRAN.R-project.org/package=caret, accessed 9 June 2021). To check for model stability and to mitigate the risk of overfitting, cross-validation steps were structured into the analysis pipeline as follows:

Both datasets (discovery, Australia; and validation, Spain) were split into two randomly allocated subgroups, resulting in a training and test set for each sample cohort (Table 1).

Training models were created using a k-fold repeated cross-validation approach (*k* = 10, repeats = 100, total *n* = 1000), resulting in 1000 cross-validated receiver operated characteristic area under the curve values (CV-ROC AUC).

The density distribution of each k-fold iteration CV-ROC AUC underwent comparison with randomly permuted test scores (*n* = 1000), which were created by randomly shuffling the sample class label for each data row, resulting in a model performance equivalent to random distribution.

This approach has been successfully employed previously in the development and testing of clinical classification models [31].

To investigate the effects of sex and age, firstly, Mann–Whitney U tests were performed to compare lipid concentrations in females and males, followed by Pearson’s correlation to investigate lipid concentration associations with age. This analysis was completed on healthy controls across the discovery and validation cohorts.

## 3. Results

### 3.1. Lipid Analysis

**Figure 1:**
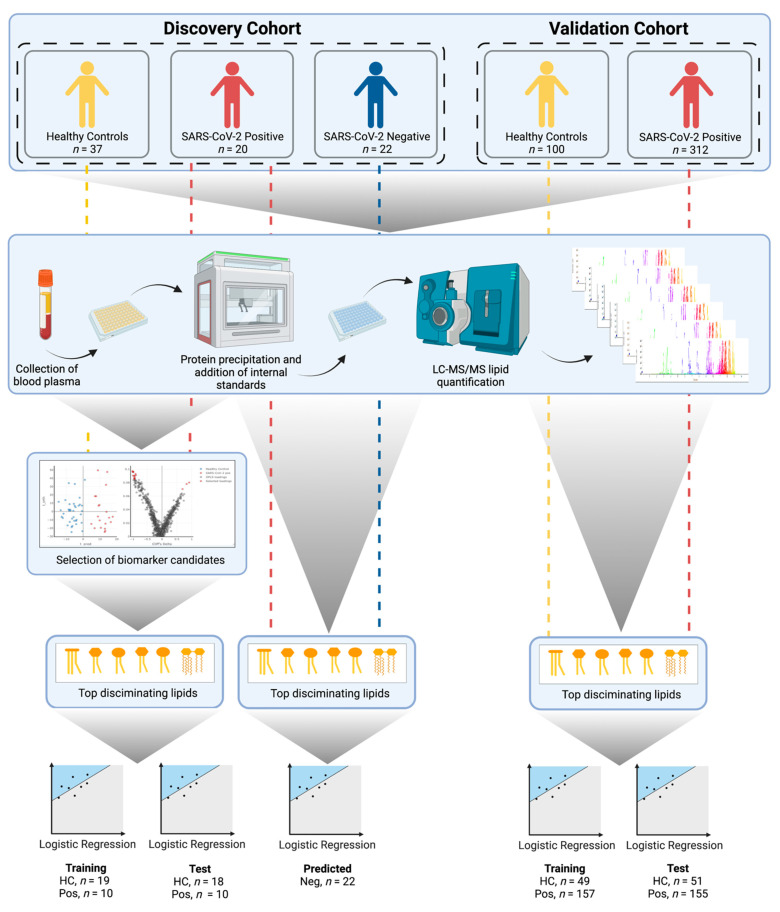
Experimental design of the study (created with BioRender.com, accessed 9 June 2021). First, lipidomic data were acquired using LC–MS. The acquired data from the discovery cohort underwent multivariate OPLS-DA modelling to facilitate the selection of biomarker candidates. A six-lipid classifier was built using logistic regression. The classifier was validated using a training/test study design, applied to two orthogonal sample collections (discovery and validation). For the discovery cohort, samples collected from individuals reporting COVID-19-like symptoms, but who tested negative for the SARS-CoV-2 virus, were projected onto the logistic regression classifier in order to evaluate the ability to differentiate between SARS-CoV-2 and other upper respiratory tract infections.

An overview of the study’s workflow is illustrated in Figure 1. Targeted lipid quantification was performed on either plasma (discovery) or serum (validation) collected from individuals who tested SARS-CoV-2 positive by nasopharyngeal PCR swab, or from healthy controls. The samples were collected from two independent cohorts: a discovery cohort (Perth, Australia) and a validation cohort (Derio, Spain). Comprehensive analysis using liquid chromatography–mass spectrometry (LC–MS) covered 19 subclasses of lipids, including triacylglycerides (TAGs), diacylglycerides (DAGs), monoacylglycerols (MAGs), free fatty acids (FFAs), sphingomyelins (SMs), ceramides (CERs), dihydroceramides (DCERs), hexosylceramides (HCERs), lactosylceramides (LCERs), cholesterol esters (CEs), phosphocholines (PCs), phosphoethanolamines (PEs), phosphoglycerols (PGs), phosphoinositols (PIs), phosphoserines (PSs), lysophosphocholines (LPCs), lysophosphoethanolamines (LPEs), lysophosphoglycerols (LPGs), and lysophosphoinositols (LPIs).

Following quality control (QC) evaluation and data filtering, LC–MS/MS analysis resulted in 845 reproducible lipids across both the discovery and validation datasets. For visualization, subsequent principal component analysis (PCA) showed that the pooled QC samples were tightly clustered, with minimal variation compared to the biological sample variance, indicating excellent reproducibility across the discovery and validation analysis (Figure S1). In addition, the PCA showed no outliers in the datasets (Figure S1).

### 3.2. Multivariate Analysis of the Discovery Cohort and Identification of Individual Lipid Species as Biomarker Candidates

**Figure 2:**
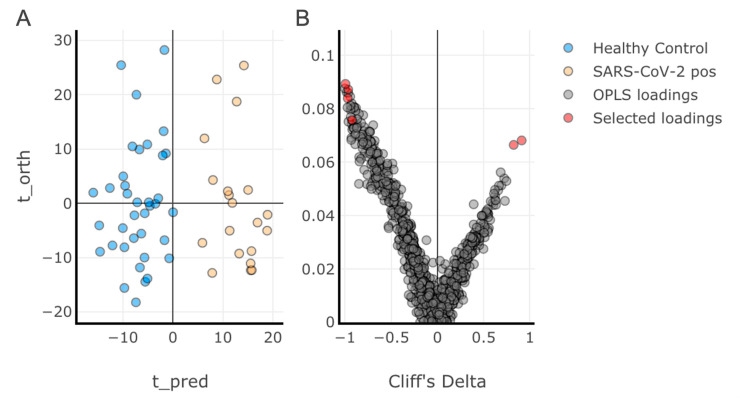
Orthogonal projection to latent structures discriminant analysis (OPLS-DA) on the discovery training dataset. (**A**) Scores plot representing the two groups (healthy controls and SARS-CoV-2-positive); (**B**) Loadings plot presented in the form of an eruption plot (Cliff’s delta vs. OPLS loadings). A discriminant model was achieved (R^2^ = 0.26, AUC = 0.99). The lipids that made up the final classifier are highlighted in panel B.

Multivariate analysis was performed using orthogonal projection to latent structures discriminant analysis (OPLS-DA) on the discovery training dataset (57 samples: 20 SARS-CoV-2-positive; 37 healthy controls; 845 lipids) (Figure 2). A discriminant model was produced (R^2^ = 0.21, CV-AUC = 0.99), indicating a high degree of dyslipidaemia with viral infection. The eruption plot highlighted the lipid species associated with SARS-CoV-2 infection. A total of 275 lipid species were found to be significantly altered between the SARS-CoV-2-positive and healthy control groups (Mann–Whitney, *p* < 0.05) (Table S5).

To select candidates for the final lipid classifier panel, lipids were filtered by the following criteria: Cliff’s delta estimates > 0.7; did not correlate with an existing member of the panel across the total dataset (Pearson’s > 0.8) (Supplementary Table S1); and only one individual lipid per class was considered. From this, a classification panel of six lipids (highlighted in the OPLS-DA loadings plot Figure 2B) were identified as fulfilling the selection criteria and having significant influence on the OPLS-DA classification, and were therefore selected for further testing as predictors of SARS-CoV-2 infection. Four of the panel were at lower concentrations in the SARS-CoV-2-positive group (PE(P-18:1/18:2), PC(18:2/18:2), LPC(18:2), and HCER(22:0)) and two were present at higher concentrations (CER(18:0) and DCER(18:0)).

Univariate analysis revealed that the six lipids were significantly different (*p* < 0.05) between healthy controls and SARS-CoV-2-positive individuals in both the discovery and validation cohorts (Table 2). The six lipids were also significantly (*p* < 0.05) different when comparing the SARS-CoV-2-negative group and the SARS-CoV-2-positive group, but were not significantly (*p* > 0.05) different when comparing lipid concentrations in SARS-CoV-2-negative individuals and healthy controls (Supplementary Table S2).

### 3.3. Diagnostic Performance of the Lipid Panel in the Discovery and Validation Cohorts

Data were randomly divided into training and test subgroups; the division between training and test was 50/50; this was performed for both the discovery and validation cohorts (Table 1).

#### 3.3.1. Discovery Cohort Training Model: SARS-CoV-2-Positive vs. Healthy Controls

**Figure 3:**
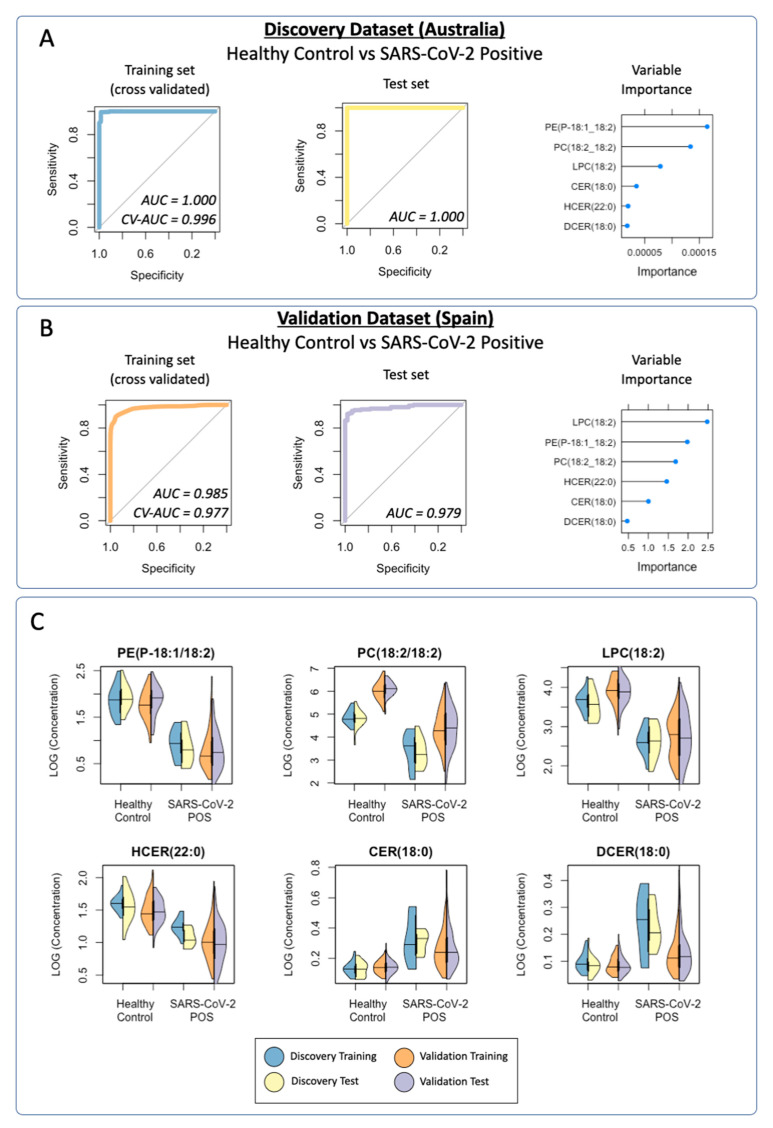
Cross-validated (CV) receiver operating characteristic (ROC) curves for the lipid logistic regression model for: (**A**) the discovery dataset (Australia) comparing SARS-CoV-2-positive individuals with healthy controls; training set (CV-AUC = 0.996, CV-sensitivity = 0.991, CV-specificity =0.909); test set (AUC = 1.000, sensitivity = 1.000, specificity = 0.944); (**B**) the validation dataset (Spain) comparing SARS-CoV-2-positive individuals with healthy controls; training set (CV-AUC = 0.977, CV-sensitivity = 0.855, CV-specificity = 0.948); test set (AUC = 0.978, sensitivity = 0.948, specificity = 0.922); (**C**) violin plots displaying the distribution of data acquired for the lipid panel. Both the discovery (training/test) and validation (training/test) cohorts demonstrated similar trends when comparing healthy controls to SARS-CoV-2-positive groups. Results of Mann–Whitney tests between the healthy control and SARS-CoV-2-positive groups for both the discovery and validation cohorts are reported in Table 2. The comparison of healthy control, SARS-CoV-2-negative, and SARS-CoV-2-positive groups can be found in Figure S2.

**Figure 4:**
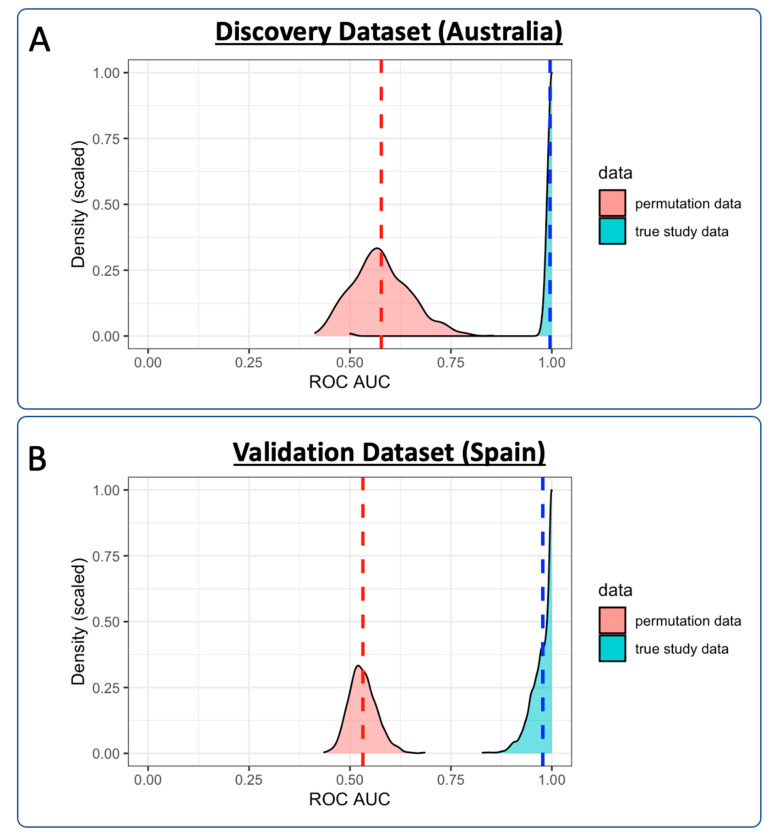
Density plot representing ROC AUC values from both of the cross-validated training models: (**A**) discovery cohort; (**B**) validation cohort. Cross-validation in the training of the models was completed using repeated *k*-fold cross-validation (*k* = 10, repeats = 100), resulting in 1000 ROC AUC scores (displayed in blue). Equivalent ROC AUC scores are displayed for permuted data (*n* = 1000) in red. Dashed lines represent ROC AUC means for the both the real study data and the permuted data.

A logistic regression classification model was trained to distinguish SARS-CoV-2-positive individuals from healthy controls in the discovery training subgroup. Cross-validation was performed using a repeated k*-*fold cross-validation approach (*k* = 10, repeats = 100, total *n* = 1000). The aggregate CV-ROC AUC = 0.996; CV-sensitivity = 0.991; and CV-specificity = 0.909 (Figure 3A). Permutation testing was performed, comparing the distribution of each CV-ROC AUC (*n* = 1000) with randomly permutated ROC AUC scores (*n* = 1000). The mean ROC AUC of the permuted data was 0.578, compared with 0.996 in the CV training data (Figure 4A). When used as a classification predictor, the final model classified all samples in the training set correctly (Table 3). Model sensitivity = 1.000; specificity = 1.000; positive predictive value = 1.000; negative predictive value = 1.000; and ROC AUC = 1.000.

#### 3.3.2. Prediction of the Discovery Cohort Test Data Using the Cross-Validated Training Model: SARS-CoV-2-Positive vs. Healthy Control

Classification prediction of the discovery test subgroup was performed using the cross-validated model. One healthy control was incorrectly classified as SARS-CoV-2-positive. All of the remaining samples were correctly classified (Table 3). Model sensitivity = 1.000; specificity = 0.944; positive prediction value = 0.909; negative prediction value: 1.000; and ROC AUC = 1.000.

#### 3.3.3. Prediction of the Discovery Cohort Test Data Using the Cross-Validated Training Model: SARS-CoV-2-Negative

Classification prediction was performed on a subgroup of participants who reported to a COVID-19 clinic with COVID-19-like symptoms but tested negative for SARS-CoV-2 infection by a PCR test. Lipid data from the SARS-CoV-2-negative group were projected onto the discovery training model. Of the 22 samples, 18 were classified as negative for SARS-CoV-2 infection, with 4 being classified as SARS-CoV-2-positive (model specificity = 0.818).

#### 3.3.4. Validation Cohort Training Model: SARS-CoV-2-Positive vs. Healthy Controls

Validation of the lipid panel was completed in an independent cohort. A logistic regression classification model was trained to distinguish SARS-CoV-2-positive individuals from healthy controls in the validation training subgroup. Again, cross-validation was performed using a repeated k*-*fold cross-validation approach (*k* = 10, repeats = 10, total *n* = 1000). The aggregate CV-ROC AUC = 0.977, CV-sensitivity = 0.855, and CV-specificity = 0.948 (Figure 3B). Permutation testing was again performed, comparing the distribution of each validation CV-ROC AUC (*n* = 1000) with randomly permutated ROC AUC scores (*n* = 1000). The mean ROC AUC of the permuted data was 0.532, compared with 0.977 in the CV training data (Figure 4B). When used as a classification predictor, the final model incorrectly classified six healthy controls and six SARS-CoV-2-positive samples (Table 3). Model sensitivity = 0.962; specificity = 0.898; positive predictive value = 0.968; negative predictive value =0.880; and ROC AUC = 0.985.

#### 3.3.5. Prediction of the Validation Cohort Test Data Using the Cross-Validated Training Model: SARS-CoV-2-Positive vs. Healthy Controls

Classification prediction of the validation test subgroup was performed using the cross-validated model. The final model incorrectly classified four healthy controls and eight SARS-CoV-2-positive samples, with all of the remaining samples being correctly classified (Table 3). Model sensitivity = 0.948; specificity = 0.922; positive prediction value = 0.974; negative prediction value: 0.855; and ROC AUC = 0.978.

#### 3.3.6. Visualization of the Distribution of the Lipid Classification Panel in SARS-CoV-2 Infection

Violin plots were created to enable visualization of each of the lipids that were included in the final six-lipid diagnostic panel (Figure 3C). PE(P-18:1/18:2), PC(18:2/18:2), LPC(18:2) and HCER(22:0) were shown to be present at lower concentrations in the SARS-CoV-2-positive groups in both the discovery and validation cohorts, whilst CER(18:0) and DCER(18:0) were present at higher concentrations in SARS-CoV-2 in both cohorts. The six lipids in the panel demonstrated remarkable similarity across the cohorts, indicating a robust diagnostic classifier.

#### 3.3.7. Effects of Age and Sex on Lipid Concentrations

Mann–Whitney U tests were completed for each lipid, comparing concentrations in males and females. No significant (*p* < 0.05) differences were observed for any of the lipids in the classification panel: PE(P-18:1/18:2), *p* = 0.18; PC(18:2/18:2), *p* = 0.15; LPC(18:2), *p* = 0.07; HCER(22:0), *p* = 0.48; CER(18:0), *p* = 0.09; and DCER(18:0), *p* = 0.08 (Table S3). Pearson’s correlation was completed for age vs. concentration of each of the lipids; no strong correlations were observed in the data: PE(P-18:1/18:2), *r* = −0.13; PC(18:2/18:2), *p* = −0.15; LPC(18:2), *r* = −0.24; HCER(22:0), *r* = 0.09; CER(18:0), *r* =0.36; and DCER(18:0), *r* =0.37 (Table S4).

## 4. Discussion

Due to the persistent threat of SARS-CoV-2 to global public health, and growing evidence of lasting health concerns associated with post-acute COVID-19 syndrome (PACS), there is a demand for more innovative and reliable biomarkers to diagnose and differentiate disease severity for personalized treatments and monitoring of convalescent patients. The present study has shown that SARS-CoV-2 infection causes significant perturbations in plasma and serum lipid profiles compared to healthy controls. Comprehensive, targeted lipid analysis revealed six lipids as major discriminating features between SARS-CoV-2-positive patients and healthy controls, which were thus regarded as potential biomarkers for the detection of SARS-CoV-2 infection. Importantly, the refined panel of lipids was validated in a second, independent cohort in order to evaluate the diagnostic potential of SARS-CoV-2 infection, and indicated robustness in the diagnostic panel, suggesting suitability for routine clinical application.

The combination of six lipids was reliably able to discriminate between SARS-CoV-2 positivity and healthy controls across two independent sample sets from different geographical locations: discovery (Perth, Australia), and validation (Derio, Spain). Furthermore, the panel also differentiated between individuals who tested positive for SARS-CoV-2, and those who reported COVID-19-like symptoms but tested negative for infection.

The lipid signatures associated with SARS-CoV-2 infection indicate profound changes in lipid metabolism and homeostasis. The candidate biomarkers contained in the SARS-CoV-2 lipid panel consisted of a heterogeneous lipid class combination, and included one each of PE, PC, LPC, HCER, CER, and DCER. The final lipid panel used in the classifier was selected using data generated via OPLS-DA. To be considered for selection, lipids required a Cliff’s delta estimate > 0.7, and could not correlate with an existing member of the panel across the total dataset (Pearson’s > 0.8). Automatic feature selection algorithms such as LASSO were avoided due to the challenges associated with model bias and their interpretation in descriptive models [32]. The lipids selected in the panel were shown to not be significantly associated with sex or age (Tables S3 and S4), indicating that the observed differences were primarily a result of the systemic response to SARS-CoV-2 infection.

The multivariate analysis in the discovery dataset indicated a global downregulation of glycerophospholipids in SARS-CoV-2 patients, including significantly lower concentrations of phosphatidylethanolamines (PEs), phosphatidylcholines (PCs), and phosphatidylglycerols (PGs)—specifically, PE(P-18:2/18:2), PE(P-16:0/18:3), PE(P-18:1/18:2), PC(18:2/20:2), PC(18:2/18:2), PC(18:2/20:1), PG(20:0/18:2), and PG(20:0/20:3), of which PE(P-18:1/18:2) was selected as the final classifier. Perturbations in phospholipids have also been implicated in the inflammatory response in acute respiratory distress syndrome, including polyunsaturated phosphatidylcholines (PUFA-PCs) and polyunsaturated phosphatidylethanolamines (PUFA-PEs) [33]. Reductions in plasma PEs and phosphatidylserines (PSs) were observed in fatal cases of Ebola virus disease [15]. In contrast, different viral infections give rise to different disease-associated changes in lipid profiles—for example, increases in serum plasmalogen PEs (PE(P)s) were observed in Zika-virus-infected patients [34], and increases in (PE(P)s) and (PC(P)s) were detected in mice administered with respiratory syncytial virus [35]. Such differences indicate that the combination of individual lipids that form the discriminatory panel may be a unique signature of SARS-CoV-2 infection, and be able to distinguish between different viral pathogens. This is reinforced in the data presented here, with significant lipid differences present when comparing SARS-CoV-2-positive individuals with the subgroup of individuals who reported COVID-19-like symptoms, indicating respiratory infection, but were negative for the virus. This indicates that SARS-CoV-2 infection may have a lipid signature that differentiates from alternative community infections.

Phospholipids are typically converted to lysophospholipids (LPs) by a family of phospholipase enzymes, including phospholipase A1, A2, and B (PLA1/2, PLAB), with multiple isoforms of each subtype. The enzymatic activities of various PLA2 enzymes in viral infections are directly associated with proinflammatory pathways, virus entry, pathogenesis, and replication [36]. The multivariate data reported the importance of LPs including LysoPCs (LPC(18:1), LPC(18:2), LPC(20:2), LPC(20:0)) and LysoPEs (LPE(18:1), LPE(18:2)) in SARS-CoV-2, with LPC(18:2) selected for the classifier panel. The lysophospholipids were found to be significantly lower compared with healthy controls in both the discovery and validation cohorts. Interestingly, this contrasts with the recent findings of Song et al., who report increases in lysophospholipids (LPAs, LPIs, and LPCs), again further reinforcing the requirement for validation in larger clinical cohorts. LPs act as second messenger molecules, modulating intracellular signalling pathways that are implicated in many biological functions, including inflammation. LPCs promote inflammatory effects, including increased endothelial expression of adhesion molecules and growth factors, monocyte chemotaxis, and macrophage activation. LPCs have also been implicated in the pathogenesis of atherosclerosis [37] and autoimmune disease [38], which have recently been implicated as risk conditions in the condition termed PACS [39].

Three sphingolipids associated with ceramide metabolism were among the most discriminant features in study, with ceramide CER(18:0), dihydroceramide DCER(18:0), and hexosylceramide HCER(22:0) selected for the classification panel. CER(18:0) and DCER(18:0) were elevated in SARS-CoV-2-infected patients compared with healthy controls, while HCER(22:0) was decreased. Ceramides are fundamental structural components of cell membranes involved in the regulation of diverse biological processes, including cell growth, differentiation, and apoptosis. Ceramides act as intracellular second messengers in diverse cell signalling pathways, including the regulation of immune cell functions. Several studies have highlighted the role of ceramide biosynthesis in response to viral infections and disease manifestation, including significant elevation of ceramides associated with proinflammatory molecules in the pathogenesis of critical infectious illnesses, including HIV [40], Ebola virus disease [15], and sepsis [41]. The abundance of total ceramide lipids was shown to be increased in fatal cases of Ebola virus disease compared with Ebola virus disease survivors—in particular, CER(16:0) and CER(18:0)—whilst plasma levels of ceramides CER(16:0) and CER(22:0) have been reported to be significantly elevated following HIV infection, with CER(16:0) closely correlating with immune activation and inflammation [40].

Ceramides have recently been shown to promote vasoconstriction, and have been implicated with cardiovascular and lung disease [42]. CER(16:0), CER(18:0), and CER(20:0), in particular, have been highly correlated with adverse cardiovascular events, atherosclerosis, and metabolic disorders [43]. In sepsis patients, ceramides have been identified as possible markers to predict multiorgan dysfunction [44]—which bears relevance to SARS-CoV-2, where multiorgan effects have been associated with infection [45]. Significant increases in CER species together with decreases in LPC species, as observed here, are a trend also detected in sepsis patients, and have shown strong predictive power for sepsis-related mortality [41]. Our data support a role for ceramides in the pathophysiology of COVID-19, with systemic inflammation making them potential diagnostic and therapeutic targets. Indeed, acid sphingomyelinase inhibition of ceramide synthesis has been shown to reduce organ damage due to reactive oxygen species and inflammation in murine models of sepsis [46], and in cystic fibrosis [47].

Finally, from the OPLS-DA multivariate model, an increase in acylglycerides (MAG, DAG, and TAG) was associated with SARS-CoV-2 (Table S5). Currently, there are no reports of MAG associations specifically with SARS-CoV-2 infection; however, the enzyme monoacylglycerol lipase (MAGL), which is the catalyst for fatty acid release from the glycerol head group, is a proinflammatory enzyme [48]. It has been reported that MAGL mice are more resistant to hepatic inflammation, reducing hepatic macrophage numbers and inflammatory gene expression, and slowing down fibrosis progression [48]. MAGL has been hypothesised as a therapeutic target in inflammatory conditions [49], and may warrant further investigation in the control of damaging inflammation caused by COVID-19.

## 5. Conclusions

Following LC–MS/MS quantitative lipidomics and multivariate data interrogation, a targeted panel of lipid biomarkers was developed and evaluated for their diagnostic potential for SARS-CoV-2 in two independent cohorts collected in different countries. Using a panel of lipids, logistic regression modelling resulted in a strong classifier model that could determine SARS-CoV-2 infection compared with healthy controls. In a subset of samples, the lipid panel was also capable of distinguishing SARS-CoV-2-positive individuals from those who reported symptoms, but tested negative for the virus, indicating that the panel may be capable of differentiating between SARS-CoV-2 and other viral infections present in the community.

This robust method demonstrates the diagnostic potential of the lipidome in infectious disease. Furthermore, not only do the results perform well in a diagnostic setting, they also provide mechanistic insight into the systemic response to infection, including the identification of perturbations in lipids associated with liver dysfunction, atherosclerosis, and autoimmune disease—all of which have been associated with post-acute COVID-19 syndrome (PACS). Hence, plasma and serum lipid concentrations may also have applications in monitoring recovery to evaluate long-term dyslipidaemia in convalescent patients.

Prior to translation to the clinic, the diagnostic capacity of the proposed lipid panel would clearly require further large-scale clinical evaluation. Evaluation in multiple cohorts from heterogeneous populations, with additional co-morbidities, would be critical to understanding the capabilities of the panel. Furthermore, additional studies are required in order to determine the precise mechanisms underlying these associations. Nevertheless, in addition to the diagnostic advantages of measuring biomarkers of the disease and improving discrimination during the early stages of infection, molecular signatures of SARS-CoV-2 provide strategies for potential therapies and personalized targets in future studies to help in optimizing precision medicine. Furthermore, biomarker panels such as these could find clinical value in stratifying infected individuals into subgroups who are at risk of severe disease, or PACS (long COVID), and provide an opportunity to track progression and recovery.

## Figures and Tables

**Table 1:** Patient samples in the discovery and validation datasets (healthy controls vs. SARS-CoV-2-positive individuals).

		Healthy	SARS-CoV-2 Pos	Sex (F/M)	Age (Std Dev)
Discovery(Australia)	Training Set	19	10	17/13	53.0 (18.4)
	Test Set	18	10	12/15	56.1 (18.1)
	All	37	20	28/29	54.5 (18.2)
Validation(Spain)	Training Set	49	157	104/102	62.9 (20.0)
	Test Set	51	155	118/88	62.3 (20.3)
	All	100	312	222/190	62.6 (20.1)
		**Training Set**	**Test Set**	**Sex (F/M)**	**Age (Std Dev)**
Discovery(Australia)	Healthy	19	18	15/22	47.54 (16.9)
	SARS-CoV-2 Pos	10	10	14/6	67.32 (12.8)
	Healthy and SARS-CoV-2 Pos	29	28	28/29	54.50 (18.2)
	SARS-CoV-2 Neg subcohort	22		13/9	47.64 (15.05)
Validation(Spain)	Healthy	49	51	50/50	42.87 (12.5)
	SARS-CoV-2 Pos	157	155	172/140	68.89 (17.9)
	Healthy and SARS-CoV-2 Pos	206	206	222/190	54.50 (18.2)

**Table 2:** Univariate analysis of the lipid classifier panel. Six candidates were selected, with four decreasing and two increasing in SARS-CoV-2. Univariate statistics for the 275 lipids that were significant in the OPLS-DA (Figure 2) can be found in Table S5.

Discovery (Australia)							
Lipid	Healthy Control Mean (sd)	SARS-CoV-2 Pos Mean (sd)	Mann–Whitney *p*	BH *q* Value	Cliff’s Delta	Cliff’s Delta 95%CI—Lower	Cliff’s Delta 95%CI—Upper
PE(P-18:1/18:2)	5.91 (2.09)	1.54 (0.84)	6.61 × 10^−15^	3.97 × 10^−14^	0.99	0.97	1.00
PC(18:2/18:2)	131.92 (46.05)	35.19 (22.5)	6.16 × 10^−13^	1.23 × 10^−12^	0.96	0.87	0.99
LPC(18:2)	37.31 (12.8)	13.9 (5.51)	3.22 × 10^−13^	9.66 × 10^−13^	0.97	0.89	0.99
HCER(22:0)	3.94 (0.89)	2.23 (0.51)	3.03 × 10^−11^	4.54 × 10^−11^	0.92	0.77	0.98
CER(18:0)	0.14 (0.05)	0.38 (0.17)	8.77 × 10^−11^	1.05 × 10^−10^	−0.91	−0.97	−0.72
DCER(18:0)	0.10 (0.04)	0.27 (0.12)	1.94 × 10^−8^	1.94 × 10^−8^	−0.82	−0.94	−0.56
**Validation (Spain)**							
**Lipid**	**Healthy Control Mean (sd)**	**SARS-CoV-2 Pos Mean (sd)**	**Mann–Whitney *p***	**BH *q* Value**	**Cliff’s Delta**	**Cliff’s Delta 95%CI—Lower**	**Cliff’s Delta 95%CI—Upper**
PE(P-18:1/18:2)	5.4 (1.99)	1.41 (1.26)	1.74 × 10^−43^	5.22 × 10^−43^	0.92	0.88	0.95
PC(18:2/18:2)	451.11 (156.5)	110.91 (100.96)	8.21 × 10^−45^	4.93 × 10^−44^	0.93	0.9	0.96
LPC(18:2)	51.23 (17.35)	17.19 (11.06)	6.38 × 10^−43^	1.28 × 10^−42^	0.91	0.86	0.94
HCER(22:0)	3.51 (0.96)	1.84 (0.92)	3.70 × 10^−35^	5.55 × 10^−35^	0.82	0.76	0.87
CER(18:0)	0.15 (0.05)	0.31 (0.18)	1.17 × 10^−22^	1.40 × 10^−22^	−0.65	−0.73	−0.56
DCER(18:0)	0.09 (0.04)	0.14 (0.09)	6.93 × 10^−9^	6.93 × 10^−9^	−0.38	−0.48	−0.28

**Table 3:** Confusion matrices for the logistic regression models in the discovery and validation data presented in Figure 3.

Discovery Dataset (SARS-CoV-2-Positive vs. Healthy Controls)				
	Training		Test	
	Healthy Control Actual	SARS-CoV-2 POS Actual	Healthy Control Actual	SARS-CoV-2 POS Actual
Model healthy control predicted	19	0	17	0
Model SARS-CoV-2 POS predicted	0	10	1	10
**Validation Dataset (SARS-CoV-2 Positive vs. Healthy Control)**				
	**Training**		**Test**	
	**Healthy Control Actual**	**SARS-CoV-2 POS Actual**	**Healthy Control Actual**	**SARS-CoV-2 POS Actual**
Model healthy control predicted	44	6	47	8
Model SARS-CoV-2 POS predicted	5	151	4	147

## Data Availability

The data presented in this study are available on request from the corresponding authors.

## Supplementary Materials

The following are available online at https://www.mdpi.com/article/10.3390/metabo11070467/s1: Figure S1: Principal component analysis of the discovery and validation datasets, Figure S2: Violin plot displaying the distribution of data acquired for the six-lipid panel from the discovery dataset, comparing healthy controls, individuals who displayed COVID-19-like symptoms but tested negative for the virus by PCR swab test (SARS-CoV-2-negative), and SARS-CoV-2-positive individuals; Table S1: Pearson’s correlation of the lipid panel across both datasets (discovery and validation), Table S2: Kruskal–Wallis univariate analysis for the lipids in the classifier panel, Table S3: Mann–Whitney U test results for each lipid, comparing female vs. male in the healthy controls of the study (including both the discovery and validation cohorts), Table S4: Pearson’s correlation values for each lipid vs. age in the healthy controls of the study (including both the discovery and validation cohorts), Table S5: Kruskal–Wallis univariate analysis for all lipids that were significant (Kruskal–Wallis p < 0.05) in the OPLS-DA model (275 lipids, Figure 2).
