# Supplementary material for: Diagnostic Potential of the Plasma Lipidome in Infectious Disease: Application to Acute SARS-CoV-2 Infection
Source: Metabolites. 2021 Jul 20;11(7):467. doi: 10.3390/metabo11070467 (PMC8306636; doi:10.3390/metabo11070467)
Supplement: Supplementary file 1 [file metabolites-11-00467-s001.zip › metabolites-1275019-supplementary.pdf]

Supplementary Data

Diagnostic Potential of the Plasma Lipidome in Infectious Disease: Application to Acute SARS-CoV-2 Infection

Nicola Gray[1,2], Nathan G. Lawler[1,2], Annie Xu Zeng[1], Monique Ryan[1], Sze How Bong[1], Berin A. Boughton[1,2], Maider Bizkarguenaga[5], Chiara Bruzzone[5], Nieves Embade[5], Julien Wist[1,3], Elaine Holmes[1,2,4], Oscar Millet[5]*, Jeremy K. Nicholson[1,2,6]*, Luke Whiley[2,7]*

[1] Australian National Phenome Centre, Health Futures Institute, Murdoch University, Harry Perkins Institute, 5 Robin Warren Drive, Perth, Western Australia 6150, Australia
[2] Centre for Computational and Systems Medicine, Health Futures Institute, Murdoch University, Harry Perkins Institute, 5 Robin Warren Drive, Perth, Western Australia 6150, Australia
[3] Chemistry Department, Universidad del Valle, 76001 Cali, Colombia
[4] Department of Metabolism Digestion and Reproduction, Faculty of Medicine, Imperial College London, Sir Alexander Fleming Building, South Kensington, London SW7 2AZ, U.K.
[5] Precision Medicine and Metabolism Laboratory, CIC bioGUNE, Basque Research and Technology Alliance, Bizkaia Science and Technology Park, building 800, 48160 Derio, Bizkaia, Spain
[6] Institute of Global Health Innovation, Imperial College London, Level 1, Faculty Building South Kensington Campus, London SW7 2AZ, U.K.
[7] Perron Institute for Neurological and Translational Science, Nedlands, Western Australia 6009, Australia

* Correspondence to luke.whiley@murdoch.edu.au, Jeremy.nicholson@murdoch.edu.au, j.nicholson@imperial.ac.uk and omillet@cicbiogune.es

Supplementary methods

Quantitative LC-MS/MS analysis

The analytical system comprised an ExionLC™ coupled to a QTRAP 6500+ system (Sciex, MA, USA). Reversed-phase separation was performed using an Acquity BEH C18 1.7 μm, 2.1 x 100 mm column (Waters Corp., MA, USA) at 60 °C. Mobile phase A was water/acetonitrile/propan-2-ol (50/30/20, v/v/v) containing 10 mM ammonium acetate and mobile phase B was propan-2-ol/acetonitrile/water (90/9/1, v/v/v) containing 10 mM ammonium acetate. The flow rate was 0.4 mL/min with gradient elution starting at 10 % B, increasing to 45 % B at 2.7 min, 53 % at 2.8 min, 60% B at 8.0 min, 80 % B at 8.1 min and holding at 80 % B until 11.5 min, 100 % B at 12.0 min for 1 min before returning to 10 % B for a 2 min re-equilibration for a total cycle time of 15 min. The injection volume was 5 μL.

A Sciex QTRAP 6500+ was operated with electrospray ionisation using polarity switching. The following mass spectrometer settings were used; capillary voltage, 5500 V (positive ion mode) and -4500 V (negative ion mode); temperature, 300 °C; curtain gas, 20 psi; ion source gas 1, 40 psi; ion source gas 2, 60 psi. Time-scheduled multiple reaction monitoring (MRM) was used for data acquisition. Data was acquired using Analyst®1.7.1.

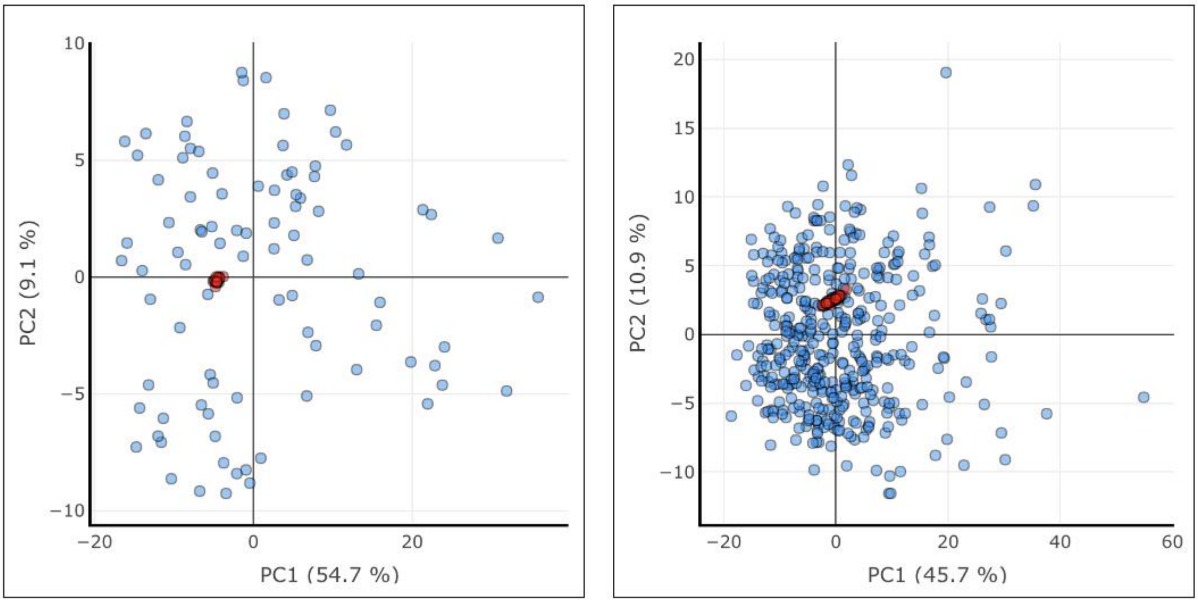

PCA of discovery dataset

PCA of validation dataset

Figure S1. *Principal component analysis of the discovery and validation datasets. Pooled QC samples are highlighted in red, with study samples in blue.*

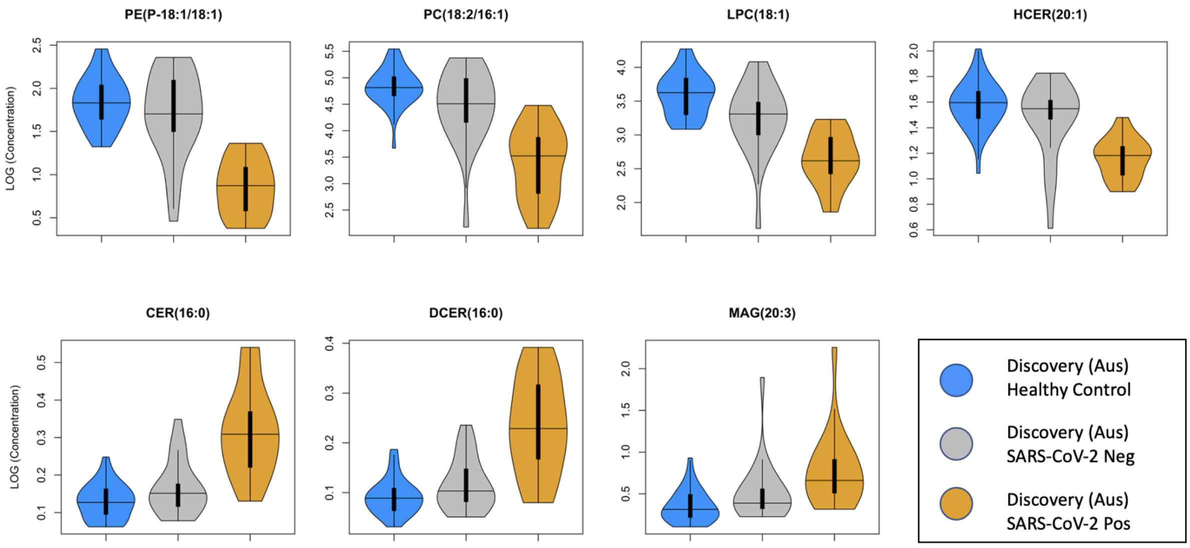

Figure S2. *Violin plot displaying the distribution of data acquired for the 6-lipid panel from the discovery dataset, comparing Healthy Control, individuals who displayed COVID-19 like symptoms but tested negative for the virus by PCR swab test (SARS-CoV-2 Neg), and SARS-CoV-2 positive.*

Table S1. *Kruskal Wallis Univariate analysis for the seven lipids in the classifier panel. Dunn's Test group comparisons were made for Healthy Control, SARS-CoV-2 negative and SARS-CoV-2 positive*

**Lipid**	**Healthy Control mean (sd)**	**SARS-CoV-2 Neg mean (sd)**	**SARS-CoV-2 Pos mean (sd)**	**Kruskal-Wallis p**	**BH q value**	**Dunn's Test p Healthy Control vs SARS-CoV-2 neg**	**Dunn's Test p Healthy Control vs SARS-CoV-2 pos**	**Dunn's Test p SARS-CoV-2 neg vs SARS-CoV-2 pos**
**PE (P-18:1/18:1)**	5.63 (1.98)	5.1 (2.59)	1.47 (0.80)	2.32E-09	7.26E-09	1.73E-01	3.80E-10	1.25E-06
**PC (18:2/16:1)**	131.87 (45.75)	104.03 (56.91)	35.16 (22.50)	3.11E-09	7.26E-09	2.65E-02	2.06E-10	4.27E-05
**LPC(18:1)**	37.34 (12.66)	27.74 (12.99)	14.02 (5.60)	2.85E-09	7.26E-09	6.54E-03	1.82E-10	2.61E-04
**HCER(20:1)**	3.93 (0.9)	3.5 (1.09)	2.22 (0.51)	9.44E-08	1.32E-07	1.14E-01	9.64E-09	3.22E-05
**CER(16:0)**	0.14 (0.05)	0.18 (0.09)	0.38 (0.17)	2.24E-08	3.92E-08	5.06E-02	1.70E-09	5.17E-05
**DCER(16:0)**	0.10 (0.04)	0.12 (0.06)	0.27 (0.12)	4.89E-07	5.71E-07	7.64E-02	4.09E-08	1.78E-04
**MAG(20:3)**	0.45 (0.31)	0.82 (1.12)	1.47 (1.77)	8.95E-06	8.95E-06	1.90E-02	7.62E-07	6.03E-03

Table S2. *Kruskal Wallis Univariate analysis for all lipids that were significant in the OPLS-DA model (**259 lipids, Figure 1**). Dunn's Test group comparisons were made for Healthy Control, SARS-CoV-2 negative and SARS-CoV-2 positive*

Lipid	Healthy Control mean (sd)	SARS-CoV-2 Neg mean (sd)	SARS-CoV-2 Pos mean (sd)	Kruskal-Wallis p	BH q value	Dunn's Test p Healthy Control vs SARS-CoV-2 neg	Dunn's Test p Healthy Control vs SARS-CoV-2 pos	Dunn's Test p SARS-CoV-2 neg vs SARS-CoV-2 pos
PE(P-18:1_18:1)	5.63 (1.98)	5.1 (2.59)	1.47 (0.8)	2.32E-09	3.88E-07	1.73E-01	3.80E-10	1.25E-06
PC(18:2_20:1)	1.01 (0.29)	0.85 (0.41)	0.36 (0.13)	2.58E-09	3.88E-07	4.83E-02	1.98E-10	1.51E-05
LPC(18:1)	37.34 (12.66)	27.74 (12.99)	14.02 (5.6)	2.85E-09	3.88E-07	6.54E-03	1.82E-10	2.61E-04
PG(20:0_18:1)	0.82 (0.19)	0.68 (0.21)	0.37 (0.09)	6.10E-10	3.66E-07	1.66E-02	3.76E-11	3.28E-05
PC(18:2_16:1)	131.87 (45.75)	104.03 (56.91)	35.16 (22.5)	3.11E-09	3.88E-07	2.65E-02	2.06E-10	4.27E-05
PE(P-16:0_18:2)	0.21 (0.1)	0.14 (0.07)	0.05 (0.03)	9.79E-10	3.66E-07	1.64E-02	6.06E-11	4.38E-05
PE(P-18:0_22:6)	0.08 (0.03)	0.07 (0.03)	0.03 (0.01)	1.36E-08	1.27E-06	4.07E-02	9.77E-10	5.38E-05
PE(P-18:1_16:0)	0.31 (0.09)	0.28 (0.11)	0.14 (0.04)	1.11E-08	1.19E-06	1.24E-01	1.28E-09	6.98E-06
PC(18:1_18:1)	147.44 (28.41)	125.18 (38.37)	79.41 (25.67)	2.44E-08	1.66E-06	2.23E-02	1.62E-09	1.81E-04
HCER(20:1)	3.93 (0.9)	3.5 (1.09)	2.22 (0.51)	9.44E-08	3.36E-06	1.14E-01	9.64E-09	3.22E-05
HCER(d18:0_20:0)	0.09 (0.02)	0.08 (0.03)	0.05 (0.01)	7.67E-08	2.87E-06	7.88E-02	6.63E-09	5.37E-05
LPC(18:3)	0.3 (0.12)	0.24 (0.09)	0.12 (0.05)	3.03E-08	1.74E-06	9.68E-02	2.94E-09	2.10E-05
PC(18:2_18:2)	2 (0.99)	1.51 (0.93)	0.56 (0.34)	5.14E-08	2.14E-06	3.22E-02	3.57E-09	1.67E-04
PC(18:2_18:3)	0.72 (0.23)	0.56 (0.21)	0.35 (0.08)	1.69E-08	1.40E-06	1.08E-02	1.11E-09	3.73E-04
PE(P-16:0_20:1)	0.47 (0.17)	0.45 (0.21)	0.14 (0.09)	3.71E-08	1.77E-06	3.12E-01	1.00E-08	1.98E-06
HCER(22:1)	6.96 (1.63)	6.34 (2.31)	3.66 (1.08)	2.16E-07	6.21E-06	1.18E-01	2.20E-08	5.12E-05
PE(P-18:0_18:2)	0.1 (0.04)	0.09 (0.03)	0.05 (0.02)	2.87E-08	1.74E-06	3.66E-02	2.02E-09	9.83E-05
HCER(d18:0_22:0)	0.15 (0.04)	0.14 (0.05)	0.08 (0.02)	2.57E-07	7.12E-06	9.82E-02	2.38E-08	7.87E-05
LPC(20:1)	0.35 (0.09)	0.29 (0.09)	0.18 (0.06)	4.62E-08	2.03E-06	2.45E-02	3.12E-09	2.30E-04

PE(P-18:1_18:2)	0.1 (0.04)	0.08 (0.03)	0.04 (0.01)	3.79E-08	1.77E-06	1.56E-02	2.52E-09	3.71E-04
PG(18:2_18:2)	0.23 (0.05)	0.2 (0.06)	0.14 (0.03)	1.33E-07	4.33E-06	2.04E-02	9.13E-09	5.33E-04
PG(20:0_18:2)	0.04 (0.01)	0.03 (0.01)	0.02 (0.01)	3.38E-08	1.77E-06	9.43E-02	3.22E-09	2.36E-05
HCER(24:1)	0.06 (0.02)	0.06 (0.02)	0.03 (0.01)	2.05E-07	6.13E-06	1.71E-01	2.65E-08	2.44E-05
LPE(18:1)	2.93 (1.16)	2.21 (1.05)	1.1 (0.58)	9.99E-08	3.40E-06	1.68E-02	6.84E-09	5.80E-04
SM(22:1)	10.88 (0.91)	10.39 (0.79)	9.24 (0.72)	7.34E-08	2.87E-06	5.41E-02	5.63E-09	9.58E-05
PE(P-18:0_20:2)	2.96 (1.09)	3.13 (1.65)	1.16 (0.54)	2.84E-07	7.59E-06	4.83E-01	1.42E-07	2.38E-06
PI(20:0_18:1)	0.3 (0.13)	0.25 (0.13)	0.11 (0.05)	1.72E-07	5.36E-06	1.11E-01	1.71E-08	4.95E-05
PI(18:1_20:4)	0.09 (0.06)	0.06 (0.05)	0.03 (0.01)	4.77E-07	1.22E-05	1.49E-02	3.52E-08	1.60E-03
PE(P-18:0_18:0)	2.02 (0.75)	1.93 (0.8)	1.02 (0.3)	1.07E-06	2.58E-05	3.08E-01	2.24E-07	2.08E-05
DCER(22:1)	2.05 (0.84)	1.95 (0.59)	1.16 (0.31)	1.19E-06	2.78E-05	4.29E-01	7.87E-07	3.91E-06
PC(18:2_20:4)	2.51 (1.62)	2.63 (2.37)	0.8 (0.67)	1.40E-06	3.08E-05	2.33E-01	2.14E-07	4.65E-05
PE(18:2_20:4)	0.04 (0.02)	0.04 (0.02)	0.02 (0.01)	2.63E-06	5.32E-05	8.59E-02	2.26E-07	4.16E-04
LPC(18:0)	28.01 (7.03)	25.18 (7.52)	16.82 (5.37)	2.47E-06	5.28E-05	1.07E-01	2.29E-07	2.83E-04
PC(18:0_18:1)	278.4 (44.22)	260.04 (64.1)	197.64 (44.52)	4.14E-06	8.00E-05	1.10E-01	3.85E-07	3.75E-04
PG(18:2_16:1)	0.05 (0.01)	0.05 (0.02)	0.04 (0.01)	4.71E-06	8.53E-05	7.63E-03	4.71E-07	1.10E-02
CE(20:1)	0.83 (0.26)	0.68 (0.32)	0.47 (0.21)	4.52E-06	8.45E-05	1.21E-02	3.99E-07	6.77E-03
PC(18:2_20:2)	3.53 (1.31)	3.47 (1.77)	1.3 (1.12)	4.17E-06	8.00E-05	4.10E-01	1.19E-06	2.68E-05
HCER(26:1)	0 (0)	0 (0)	0 (0)	1.32E-06	2.99E-05	3.13E-02	9.76E-08	1.14E-03
PC(18:2_22:6)	1.74 (0.77)	1.55 (0.61)	0.88 (0.39)	4.79E-06	8.53E-05	1.89E-01	5.88E-07	1.61E-04
PI(18:1_18:1)	1.11 (0.58)	1.06 (0.62)	0.53 (0.21)	1.66E-05	2.48E-04	2.33E-01	2.28E-06	2.49E-04
FFA(22:6)	1.44 (0.96)	1.02 (0.41)	0.84 (0.35)	2.59E-06	5.32E-05	6.40E-03	2.57E-07	9.61E-03
PE(P-16:0_22:6)	0.14 (0.05)	0.13 (0.05)	0.08 (0.02)	7.10E-06	1.24E-04	2.33E-01	1.01E-06	1.40E-04
LPC(18:2)	0.63 (0.3)	0.51 (0.24)	0.28 (0.14)	8.89E-06	1.46E-04	1.21E-01	8.53E-07	5.21E-04
PC(20:0_20:3)	2.2 (0.82)	1.72 (0.6)	1.3 (0.48)	9.56E-06	1.52E-04	1.54E-02	8.51E-07	7.81E-03

PC(20:0_18:1)	1.08 (0.45)	0.99 (0.44)	0.49 (0.32)	1.45E-05	2.21E-04	2.19E-01	1.91E-06	2.54E-04
LPE(18:0)	2.14 (0.86)	1.69 (0.66)	1.14 (0.51)	3.24E-05	4.49E-04	3.95E-02	2.74E-06	5.34E-03
PE(P-18:0_16:0)	0.06 (0.02)	0.05 (0.02)	0.04 (0.01)	2.42E-05	3.42E-04	1.01E-01	2.20E-06	1.28E-03
PI(18:0_20:0)	0.16 (0.09)	0.13 (0.07)	0.07 (0.04)	2.09E-05	3.01E-04	1.26E-01	2.02E-06	8.43E-04
LPC(16:1)	33.07 (8.23)	33.15 (8.93)	21.41 (7.36)	9.80E-06	1.53E-04	2.86E-01	1.11E-05	8.40E-06
HCER(d18:0_26:1)	0.25 (0.09)	0.23 (0.09)	0.14 (0.04)	3.46E-05	4.62E-04	3.73E-01	7.39E-06	1.53E-04
LPE(18:2)	0.07 (0.04)	0.05 (0.03)	0.03 (0.02)	6.92E-05	7.96E-04	1.05E-01	6.41E-06	2.34E-03
PC(18:1_18:3)	1.77 (0.59)	1.44 (0.48)	1.06 (0.4)	3.74E-05	4.91E-04	2.15E-02	3.48E-06	1.15E-02
PS(20:0_22:6)	16.07 (4.12)	15.59 (4.2)	10.92 (2.65)	3.34E-05	4.54E-04	3.38E-01	6.33E-06	1.86E-04
CER(22:1)	19.54 (5.07)	19.05 (5.28)	13.37 (3.78)	3.83E-05	4.94E-04	4.77E-01	1.43E-05	7.00E-05
LPC(20:0)	0.4 (0.13)	0.34 (0.1)	0.26 (0.08)	7.21E-05	8.17E-04	4.26E-02	6.36E-06	7.75E-03
LPE(18:3)	0.05 (0.02)	0.04 (0.01)	0.03 (0.01)	4.58E-05	5.81E-04	5.75E-02	3.92E-06	4.12E-03
PC(14:0_18:1)	13.14 (5.96)	11.72 (6.24)	5.68 (4.79)	5.52E-05	6.55E-04	1.76E-01	6.15E-06	9.10E-04
PC(16:0_18:1)	596.29 (124.19)	540.32 (151.71)	425.05 (126.66)	8.98E-05	9.88E-04	6.29E-02	7.92E-06	5.48E-03
PC(18:1_18:2)	3.47 (1.45)	2.99 (1.49)	1.79 (1.06)	7.72E-05	8.62E-04	1.15E-01	7.32E-06	2.21E-03
LPC(20:2)	1.84 (0.54)	1.59 (0.54)	1.08 (0.55)	1.05E-04	1.12E-03	8.16E-02	9.43E-06	4.30E-03
LPE(22:4)	0.08 (0.02)	0.07 (0.02)	0.05 (0.02)	4.72E-05	5.88E-04	3.43E-03	8.98E-06	6.72E-02
PG(16:0_18:1)	0.03 (0.02)	0.02 (0.01)	0.01 (0.01)	6.04E-05	7.06E-04	7.85E-03	8.12E-06	3.86E-02
PI(18:1_16:1)	6.05 (3.16)	5.97 (3.27)	2.56 (1.87)	5.18E-05	6.25E-04	4.77E-01	1.58E-05	1.13E-04
TAG(56:2_FA18:0)	1.26 (0.58)	0.94 (0.5)	0.62 (0.38)	1.30E-04	1.35E-03	1.90E-02	1.41E-05	2.54E-02
CE(18:1)	1077.14 (176.33)	993.96 (256.34)	754.22 (253.94)	2.45E-04	2.32E-03	1.87E-01	2.77E-05	2.20E-03
SM(24:1)	0.45 (0.15)	0.4 (0.14)	0.28 (0.1)	1.73E-04	1.73E-03	1.55E-01	1.81E-05	2.36E-03
CE(18:3)	0.05 (0.03)	0.04 (0.02)	0.02 (0.02)	2.06E-04	2.02E-03	1.34E-01	2.07E-05	3.27E-03
PI(18:0_18:0)	3.06 (1.22)	3.02 (1.48)	1.77 (0.74)	3.27E-04	2.98E-03	3.62E-01	5.95E-05	8.16E-04

SM(22:0)	19.39 (3.35)	18.54 (3.43)	15.31 (2.91)	2.63E-04	2.46E-03	2.47E-01	3.46E-05	1.45E-03
LPC(22:4)	0.13 (0.05)	0.1 (0.03)	0.08 (0.03)	2.08E-04	2.02E-03	4.54E-02	1.96E-05	1.32E-02
LCER(22:1)	0.67 (0.2)	0.66 (0.18)	0.49 (0.14)	2.11E-04	2.02E-03	3.88E-01	9.84E-05	1.64E-04
DCER(22:0)	0.53 (0.17)	0.53 (0.25)	0.32 (0.19)	4.00E-04	3.52E-03	3.91E-01	7.88E-05	8.05E-04
LCER(d18:0_22:0)	0.01 (0)	0.01 (0)	0.01 (0)	1.58E-04	1.62E-03	3.26E-01	1.01E-04	9.56E-05
HCER(18:1)	0.44 (0.13)	0.41 (0.14)	0.31 (0.1)	1.09E-03	8.86E-03	1.88E-01	1.25E-04	5.90E-03
PC(18:1_20:1)	0.99 (0.39)	0.95 (0.44)	0.64 (0.26)	1.46E-03	1.14E-02	3.25E-01	2.27E-04	2.93E-03
FFA(24:1)	0.02 (0.01)	0.02 (0.01)	0.01 (0)	1.20E-04	1.26E-03	3.01E-01	9.12E-05	6.75E-05
LCER(d18:0_24:1)	2.69 (0.76)	2.87 (1.08)	1.63 (0.96)	2.88E-04	2.66E-03	2.71E-01	2.32E-04	1.18E-04
CE(14:0)	385.98 (94.64)	336.14 (112.7)	289.82 (90.74)	2.57E-03	1.81E-02	2.79E-02	3.73E-04	8.66E-02
DCER(24:1)	0.03 (0.02)	0.03 (0.01)	0.02 (0.01)	3.17E-03	2.06E-02	2.06E-01	3.82E-04	1.05E-02
PG(20:0_20:3)	0.36 (0.08)	0.3 (0.08)	0.27 (0.08)	7.84E-04	6.52E-03	4.41E-03	2.41E-04	1.97E-01
TAG(54:1_FA18:1)	2.12 (1.13)	1.64 (1.01)	1.2 (1.07)	2.52E-03	1.81E-02	5.48E-02	2.93E-04	4.52E-02
PC(14:0_20:4)	0.37 (0.14)	0.34 (0.24)	0.22 (0.25)	1.61E-03	1.23E-02	1.38E-01	1.72E-04	1.18E-02
PC(16:0_18:3)	3.69 (1.27)	3.41 (1.48)	2.53 (0.99)	2.49E-03	1.81E-02	1.34E-01	2.70E-04	1.61E-02
PI(18:2_18:2)	0.55 (0.19)	0.46 (0.15)	0.39 (0.11)	2.89E-03	1.97E-02	4.62E-02	3.58E-04	5.78E-02
HCER(d18:0_18:0)	0.01 (0)	0.01 (0)	0.01 (0)	2.81E-03	1.94E-02	4.89E-01	6.74E-04	2.15E-03
PC(18:0_20:1)	23.84 (9.94)	24.65 (12.31)	14.95 (6.96)	3.15E-03	2.06E-02	4.76E-01	7.21E-04	2.49E-03
PS(20:0_18:1)	30.89 (10.01)	28.53 (13.39)	20.75 (12.23)	3.01E-03	2.01E-02	1.43E-01	3.33E-04	1.67E-02
DCER(26:0)	0.06 (0.03)	0.06 (0.04)	0.04 (0.02)	3.67E-03	2.33E-02	3.17E-01	5.52E-04	5.94E-03
PC(16:0_20:1)	6.33 (2.82)	5.99 (2.88)	4.18 (1.65)	3.24E-03	2.09E-02	3.08E-01	4.78E-04	5.70E-03
LPC(20:5)	0.07 (0.02)	0.06 (0.03)	0.05 (0.02)	1.21E-02	5.95E-02	4.75E-02	1.89E-03	1.26E-01
PC(16:1_18:2)	0.18 (0.08)	0.19 (0.1)	0.11 (0.07)	2.56E-03	1.81E-02	4.26E-01	8.19E-04	1.39E-03

PE(P-18:0_20:3)	13.68 (5.15)	13.4 (6.72)	9.53 (3.54)	9.40E-03	4.92E-02	1.51E-01	1.13E-03	3.27E-02
LCER(20:0)	0.12 (0.04)	0.12 (0.03)	0.09 (0.03)	2.27E-03	1.70E-02	2.78E-01	1.38E-03	6.82E-04
PC(18:1_22:6)	9.34 (3.7)	8.14 (3.46)	6.4 (4.18)	7.97E-03	4.52E-02	1.24E-01	9.40E-04	3.70E-02
PS(20:0_18:2)	1.2 (0.45)	1.06 (0.5)	0.89 (0.84)	5.74E-03	3.49E-02	1.31E-01	6.59E-04	2.82E-02
CER(24:1)	0.37 (0.17)	0.35 (0.13)	0.24 (0.14)	6.16E-03	3.69E-02	4.98E-01	1.46E-03	3.81E-03
FFA(18:3)	0.71 (0.27)	0.66 (0.38)	0.53 (0.12)	1.11E-02	5.57E-02	6.55E-02	1.52E-03	8.91E-02
PE(18:1_18:1)	5.38 (4.2)	4.27 (2.36)	3.17 (2.94)	8.48E-03	4.68E-02	2.17E-01	1.07E-03	1.90E-02
PG(18:1_20:4)	0.19 (0.03)	0.18 (0.04)	0.17 (0.03)	1.03E-02	5.24E-02	5.74E-02	1.45E-03	9.67E-02
LPC(22:6)	1.34 (0.35)	1.23 (0.41)	1.04 (0.45)	1.45E-02	6.74E-02	1.24E-01	1.82E-03	5.40E-02
PC(18:1_20:4)	6.86 (3.57)	7.44 (5.06)	4.39 (3.1)	9.54E-03	4.92E-02	4.22E-01	1.81E-03	7.29E-03
PI(18:1_18:2)	0.74 (0.26)	0.64 (0.26)	0.52 (0.34)	8.85E-03	4.80E-02	9.62E-02	1.08E-03	5.26E-02
CE(22:6)	0.02 (0)	0.02 (0)	0.01 (0.01)	1.12E-02	5.59E-02	1.87E-01	1.39E-03	2.79E-02
LPC(14:0)	135.57 (27.19)	135.84 (38.99)	102.56 (36.43)	7.83E-03	4.47E-02	4.48E-01	2.16E-03	3.71E-03
PC(14:0_14:0)	6.35 (2.37)	6.66 (3.68)	4.08 (3.32)	9.35E-03	4.92E-02	3.91E-01	1.65E-03	8.25E-03
PC(14:0_18:3)	0.85 (0.48)	0.9 (0.65)	0.5 (0.57)	1.23E-02	6.01E-02	4.62E-01	2.57E-03	7.52E-03
PC(18:1_22:5)	7.65 (2.83)	6.52 (2.75)	5.65 (1.65)	1.35E-02	6.51E-02	2.76E-02	2.85E-03	2.08E-01
TAG(50:1_FA18:1)	0.26 (0.18)	0.2 (0.18)	0.13 (0.1)	1.68E-02	7.66E-02	8.06E-02	2.33E-03	9.32E-02
TAG(52:0_FA18:0)	0.73 (0.52)	0.5 (0.38)	0.43 (0.58)	8.56E-03	4.68E-02	5.09E-02	1.22E-03	9.74E-02
PC(14:0_20:3)	2.7 (1.09)	2.6 (1.74)	1.77 (1.71)	1.32E-02	6.41E-02	1.90E-01	1.67E-03	3.07E-02
PC(18:0_18:2)	2.64 (1.43)	2.64 (1.39)	1.73 (0.66)	1.60E-02	7.34E-02	4.67E-01	3.36E-03	9.08E-03
PC(18:0_22:6)	47.66 (12.43)	45.38 (14.03)	36.96 (14.48)	1.87E-02	8.33E-02	2.02E-01	2.45E-03	3.59E-02
PE(18:0_22:6)	0.57 (0.31)	0.45 (0.25)	0.35 (0.18)	1.42E-02	6.72E-02	5.05E-02	2.24E-03	1.30E-01
PI(18:0_18:1)	34.04 (17.92)	31.96 (17.41)	21.68 (10.06)	2.14E-02	9.42E-02	3.10E-01	3.24E-03	2.20E-02
TAG(56:6_FA18:	0.31 (0.2)	0.21 (0.14)	0.18 (0.08)	1.16E-02	5.75E-02	1.36E-02	3.79E-03	3.18E-01

3)								
PG(18:1_16:1)	0.11 (0.02)	0.11 (0.03)	0.09 (0.03)	3.62E-02	1.37E-01	2.01E-01	5.03E-03	5.70E-02
CE(18:2)	337.28 (128.92)	318.78 (160.44)	266.18 (229.73)	2.19E-02	9.52E-02	2.39E-01	3.00E-03	3.21E-02
CE(22:4)	2.23 (0.57)	2.08 (0.86)	1.8 (0.69)	2.60E-02	1.09E-01	6.63E-02	4.20E-03	1.45E-01
TAG(48:4_FA18:1)	3.85 (4.19)	1.81 (1.49)	1.7 (1.79)	1.76E-02	7.98E-02	2.60E-02	4.18E-03	2.50E-01
TAG(48:5_FA18:2)	0.36 (0.45)	0.16 (0.13)	0.18 (0.25)	1.41E-02	6.72E-02	1.61E-02	4.33E-03	3.12E-01
HCER(26:0)	0.04 (0.01)	0.04 (0.01)	0.03 (0.01)	3.89E-02	1.46E-01	4.94E-01	8.63E-03	1.68E-02
PC(18:1_20:2)	18.9 (5.2)	18.47 (7.12)	13.89 (9.26)	4.18E-02	1.53E-01	4.34E-01	8.11E-03	2.20E-02
PI(18:0_20:4)	0.17 (0.06)	0.17 (0.08)	0.13 (0.06)	4.07E-02	1.52E-01	2.46E-01	5.89E-03	4.80E-02
TAG(47:2_FA18:1)	0.94 (0.8)	0.58 (0.58)	0.56 (0.79)	2.49E-02	1.06E-01	3.53E-02	5.45E-03	2.38E-01
PC(16:0_16:1)	3.99 (1.09)	4.08 (1.23)	3.3 (0.96)	3.04E-02	1.23E-01	3.84E-01	9.58E-03	9.11E-03
PI(16:0_20:2)	0.63 (0.36)	0.68 (0.29)	0.43 (0.32)	9.46E-03	4.92E-02	1.10E-01	1.53E-02	1.30E-03
TAG(44:2_FA18:1)	2.28 (2.62)	1.08 (1.57)	1.08 (1.83)	2.71E-02	1.13E-01	3.23E-02	6.44E-03	2.66E-01
TAG(46:3_FA18:3)	0.57 (0.7)	0.24 (0.26)	0.26 (0.37)	2.45E-02	1.05E-01	2.34E-02	7.07E-03	3.19E-01
PC(14:0_22:6)	2.36 (1.09)	2.61 (1.53)	1.71 (1.18)	2.98E-02	1.22E-01	3.99E-01	8.96E-03	9.41E-03
PE(18:1_18:2)	0.38 (0.35)	0.23 (0.14)	0.2 (0.18)	3.11E-02	1.23E-01	5.70E-02	5.59E-03	1.84E-01
TAG(46:2_FA18:1)	5.87 (5.57)	3.75 (4.45)	3.09 (3.93)	3.32E-02	1.29E-01	6.02E-02	5.92E-03	1.82E-01
TAG(46:3_FA18:1)	2.35 (2.22)	1.35 (1.73)	1.33 (1.89)	2.94E-02	1.21E-01	2.91E-02	7.77E-03	3.01E-01
TAG(56:5_FA18:2)	0.47 (0.33)	0.32 (0.22)	0.27 (0.16)	3.12E-02	1.23E-01	3.45E-02	7.46E-03	2.74E-01
LCER(26:1)	0.01 (0)	0.01 (0)	0.01 (0)	7.52E-03	4.34E-02	1.09E-01	1.27E-02	1.03E-03
TAG(44:2_FA18:2)	0.5 (0.52)	0.28 (0.49)	0.28 (0.45)	3.11E-02	1.23E-01	2.49E-02	9.41E-03	3.44E-01
TAG(50:5_FA18:1)	2.22 (2.19)	1.32 (1.01)	1.3 (1.33)	4.14E-02	1.53E-01	4.64E-02	9.06E-03	2.55E-01

TAG(51:4_FA20:4)	0.54 (0.47)	0.32 (0.21)	0.31 (0.27)	2.96E-02	1.22E-01	3.80E-02	6.55E-03	2.47E-01
TAG(54:8_FA18:2)	1.04 (1.65)	0.53 (0.59)	0.41 (0.4)	4.21E-02	1.53E-01	5.90E-02	8.09E-03	2.12E-01
LPC(20:3)	1.64 (0.54)	1.4 (0.51)	1.34 (0.39)	4.50E-02	1.62E-01	2.70E-02	1.51E-02	3.94E-01
TAG(46:3_FA18:2)	0.6 (0.63)	0.32 (0.34)	0.37 (0.56)	3.39E-02	1.31E-01	2.83E-02	9.64E-03	3.30E-01
TAG(48:4_FA20:4)	0.46 (0.56)	0.21 (0.18)	0.24 (0.35)	3.43E-02	1.32E-01	2.73E-02	1.01E-02	3.40E-01
TAG(56:3_FA18:2)	0.66 (0.56)	0.45 (0.47)	0.33 (0.26)	4.60E-02	1.65E-01	4.31E-02	1.10E-02	2.87E-01
TAG(50:5_FA18:2)	1.73 (1.62)	1.05 (0.72)	1.29 (1.58)	4.51E-02	1.62E-01	3.63E-02	1.20E-02	3.22E-01
CE(22:2)	0.41 (0.13)	0.34 (0.16)	0.34 (0.11)	3.54E-02	1.35E-01	1.11E-02	2.55E-02	4.06E-01
LPE(20:3)	0.5 (0.16)	0.38 (0.11)	0.41 (0.17)	8.36E-03	4.67E-02	1.96E-03	1.86E-02	2.61E-01
TAG(51:5_FA18:2)	0.38 (0.31)	0.23 (0.15)	0.27 (0.27)	4.16E-02	1.53E-01	2.62E-02	1.37E-02	3.86E-01
DAG(18:2_22:5)	0.03 (0.01)	0.04 (0.01)	0.03 (0.03)	1.46E-02	6.74E-02	4.59E-02	5.51E-02	1.84E-03
TAG(58:6_FA18:1)	0.11 (0.04)	0.1 (0.05)	0.14 (0.05)	2.75E-02	1.14E-01	1.08E-01	3.83E-02	3.82E-03
TAG(58:7_FA20:4)	0.16 (0.09)	0.13 (0.09)	0.2 (0.07)	9.48E-03	4.92E-02	1.15E-01	1.45E-02	1.32E-03
TAG(56:6_FA20:5)	0.57 (0.37)	0.55 (0.4)	0.82 (0.42)	2.45E-02	1.05E-01	3.00E-01	1.07E-02	5.80E-03
DAG(18:0_18:3)	0.01 (0.01)	0.01 (0.01)	0.02 (0.01)	3.12E-02	1.23E-01	6.48E-02	5.29E-03	1.65E-01
TAG(52:3_FA16:0)	14.32 (6.14)	16.02 (8.65)	20.65 (10.83)	4.22E-02	1.53E-01	3.36E-01	6.84E-03	3.24E-02
TAG(56:7_FA20:3)	3.06 (1.22)	2.83 (1.48)	4 (1.44)	5.94E-03	3.58E-02	1.19E-01	9.43E-03	8.52E-04
TAG(58:7_FA18:2)	0.09 (0.04)	0.09 (0.06)	0.12 (0.05)	3.26E-02	1.27E-01	4.70E-01	7.95E-03	1.28E-02
DAG(18:0_22:6)	16.01 (7.82)	18.01 (10.84)	25.71 (15.14)	3.21E-02	1.26E-01	2.84E-01	4.76E-03	3.35E-02
TAG(56:4_FA22:4)	2.21 (1.84)	3.18 (4)	4.86 (4.76)	4.17E-02	1.53E-01	3.81E-01	7.28E-03	2.67E-02
DAG(16:0_16:0)	0.32 (0.21)	0.56 (0.78)	0.72 (0.74)	3.59E-02	1.36E-01	1.53E-01	4.97E-03	7.73E-02

TAG(54:4_FA20:4)	0.23 (0.2)	0.35 (0.5)	0.43 (0.31)	3.21E-02	1.26E-01	4.28E-01	6.13E-03	1.83E-02
TAG(56:6_FA16:0)	0.32 (0.18)	0.32 (0.19)	0.43 (0.17)	1.81E-02	8.16E-02	4.56E-01	4.68E-03	7.54E-03
TAG(58:6_FA18:0)	0.84 (0.61)	1.02 (1.04)	1.5 (1.2)	2.18E-02	9.52E-02	3.89E-01	6.89E-03	6.99E-03
DAG(16:1_22:6)	1.31 (0.93)	1.93 (1.7)	2.31 (1.68)	3.07E-02	1.23E-01	1.09E-01	4.37E-03	1.00E-01
DAG(16:0_22:5)	0.1 (0.07)	0.16 (0.14)	0.17 (0.12)	2.28E-02	9.86E-02	4.94E-02	4.06E-03	1.74E-01
DCER(24:0)	0.45 (0.23)	0.46 (0.24)	0.69 (0.33)	2.56E-02	1.08E-01	4.65E-01	5.32E-03	1.33E-02
DAG(18:2_20:3)	0.19 (0.09)	0.22 (0.17)	0.29 (0.14)	7.35E-03	4.30E-02	4.80E-01	1.84E-03	3.99E-03
DAG(18:1_22:5)	0.32 (0.21)	0.46 (0.34)	0.57 (0.38)	1.86E-02	8.33E-02	6.90E-02	2.76E-03	1.15E-01
SM(16:0)	21.89 (3.8)	22.43 (3.42)	25.46 (4.93)	1.09E-02	5.51E-02	2.70E-01	1.51E-03	1.66E-02
TAG(52:4_FA20:3)	1.61 (1.14)	2.16 (2.26)	2.82 (2.06)	2.13E-02	9.42E-02	2.69E-01	3.02E-03	2.68E-02
TAG(56:5_FA20:4)	1.25 (0.84)	1.42 (1.25)	2.25 (1.47)	1.41E-02	6.72E-02	4.83E-01	3.09E-03	7.70E-03
PG(18:0_18:2)	0.13 (0.02)	0.13 (0.03)	0.14 (0.02)	1.43E-02	6.73E-02	1.46E-01	1.78E-03	4.46E-02
PI(20:0_18:2)	6.31 (3.81)	7.99 (4.28)	11.94 (8.8)	7.54E-03	4.34E-02	5.23E-02	1.04E-03	8.84E-02
TAG(58:7_FA22:6)	0.96 (0.76)	1.13 (0.88)	1.74 (1.13)	9.85E-03	5.05E-02	2.87E-01	1.40E-03	1.41E-02
TAG(60:10_FA22:5)	0.13 (0.08)	0.17 (0.13)	0.21 (0.1)	1.44E-02	6.73E-02	2.20E-01	1.89E-03	2.69E-02
CER(14:0)	0.36 (0.08)	0.36 (0.05)	0.47 (0.14)	4.69E-03	2.90E-02	4.25E-01	9.14E-04	4.21E-03
DAG(18:1_20:5)	0.05 (0.03)	0.07 (0.06)	0.11 (0.08)	8.32E-03	4.67E-02	2.22E-01	1.06E-03	1.81E-02
DCER(18:0)	0.08 (0.02)	0.09 (0.02)	0.13 (0.05)	7.22E-03	4.29E-02	3.09E-01	1.07E-03	1.01E-02
PE(O-18:0_18:2)	0.55 (0.2)	0.62 (0.33)	0.85 (0.38)	8.57E-03	4.68E-02	2.85E-01	1.21E-03	1.29E-02
TAG(54:4_FA22:4)	4.3 (3.12)	5.73 (5.2)	8.93 (7.4)	9.25E-03	4.92E-02	1.90E-01	1.14E-03	2.42E-02
TAG(54:6_FA20:3)	4.72 (2.21)	4.92 (2.74)	7.98 (4.52)	3.92E-03	2.46E-02	4.44E-01	8.11E-04	3.38E-03
TAG(58:8_FA20:4)	0.27 (0.11)	0.25 (0.13)	0.38 (0.13)	2.37E-03	1.76E-02	2.59E-01	1.58E-03	6.55E-04

DAG(16:0_20:5)	0.06 (0.04)	0.09 (0.08)	0.12 (0.07)	7.31E-03	4.30E-02	1.80E-01	8.81E-04	2.22E-02
TAG(58:7_FA16:0)	0.2 (0.17)	0.28 (0.24)	0.36 (0.24)	9.10E-03	4.90E-02	9.18E-02	1.12E-03	5.63E-02
DAG(16:1_20:2)	0.04 (0.02)	0.06 (0.06)	0.09 (0.08)	5.14E-03	3.15E-02	1.77E-01	6.06E-04	1.79E-02
DAG(16:1_20:4)	0.03 (0.02)	0.06 (0.05)	0.08 (0.07)	4.16E-03	2.59E-02	4.27E-02	5.56E-04	7.63E-02
MAG(18:0)	2.43 (1.63)	4.81 (7.71)	6.31 (7.89)	1.18E-03	9.49E-03	6.08E-03	3.26E-04	1.90E-01
TAG(58:6_FA20:4)	0.31 (0.18)	0.3 (0.22)	0.51 (0.24)	1.45E-03	1.14E-02	2.92E-01	8.64E-04	4.95E-04
TAG(55:5_FA18:2)	0.25 (0.12)	0.3 (0.23)	0.5 (0.34)	2.78E-03	1.94E-02	3.38E-01	4.41E-04	4.35E-03
CER(24:0)	6.89 (1.71)	7.31 (2.1)	9.51 (3.01)	2.99E-03	2.01E-02	3.33E-01	4.67E-04	4.71E-03
DAG(18:2_22:4)	0.02 (0.01)	0.03 (0.02)	0.04 (0.02)	1.88E-03	1.42E-02	4.39E-01	3.95E-04	1.98E-03
TAG(54:5_FA20:3)	7.23 (3.24)	8 (4.42)	12.21 (6.43)	2.57E-03	1.81E-02	3.07E-01	3.80E-04	4.86E-03
TAG(58:9_FA22:6)	0.03 (0.02)	0.04 (0.02)	0.06 (0.02)	3.03E-03	2.01E-02	3.46E-01	4.88E-04	4.45E-03
DAG(16:0_20:3)	0.14 (0.09)	0.19 (0.19)	0.27 (0.17)	2.82E-03	1.94E-02	1.71E-01	3.22E-04	1.26E-02
DAG(18:2_20:5)	0.01 (0.01)	0.01 (0.01)	0.01 (0.01)	1.26E-03	1.00E-02	4.63E-01	2.92E-04	1.31E-03
LPI(20:3)	0.13 (0.04)	0.15 (0.06)	0.17 (0.04)	3.42E-03	2.19E-02	9.34E-02	3.78E-04	3.04E-02
HCER(18:0)	0.01 (0)	0.01 (0)	0.01 (0.01)	9.47E-04	7.78E-03	6.66E-02	9.65E-05	2.06E-02
TAG(54:4_FA20:3)	0.74 (0.36)	0.86 (0.55)	1.45 (0.91)	1.54E-03	1.19E-02	2.09E-01	1.84E-04	6.31E-03
PE(18:0_20:3)	9.29 (3.63)	10.36 (5.31)	16.1 (7.3)	6.63E-04	5.57E-03	2.71E-01	9.14E-05	2.32E-03
TAG(58:8_FA20:3)	0.23 (0.11)	0.25 (0.15)	0.45 (0.23)	4.82E-04	4.10E-03	4.07E-01	9.89E-05	8.48E-04
DAG(18:1_20:3)	0.46 (0.2)	0.57 (0.36)	0.94 (0.55)	4.28E-04	3.71E-03	2.11E-01	5.10E-05	2.64E-03
TAG(56:4_FA20:3)	0.21 (0.14)	0.34 (0.41)	0.5 (0.39)	4.32E-04	3.71E-03	2.51E-01	5.69E-05	1.98E-03
TAG(56:5_FA16:0)	1.03 (0.58)	1.21 (0.87)	1.83 (0.92)	3.38E-04	3.03E-03	3.14E-01	5.33E-05	1.11E-03
TAG(56:6_FA20:3)	4.32 (1.66)	4.26 (1.8)	7.11 (2.77)	1.64E-04	1.66E-03	4.35E-01	6.42E-05	1.69E-04

DAG(18:1_22:4)	0.11 (0.06)	0.15 (0.12)	0.25 (0.14)	3.40E-04	3.03E-03	1.93E-01	3.89E-05	2.61E-03
TAG(56:5_FA20:3)	2.91 (1.38)	3.13 (1.81)	5.59 (2.74)	9.55E-05	1.04E-03	4.02E-01	2.12E-05	2.68E-04
TAG(58:7_FA22:4)	0.32 (0.13)	0.33 (0.19)	0.56 (0.2)	4.93E-05	6.05E-04	4.99E-01	1.64E-05	9.66E-05
CER(20:0)	0.01 (0)	0.01 (0)	0.02 (0.01)	1.83E-05	2.68E-04	1.62E-01	1.98E-06	5.09E-04
MAG(20:3)	0.45 (0.31)	0.82 (1.12)	1.47 (1.77)	8.95E-06	1.46E-04	1.90E-02	7.62E-07	6.03E-03
CER(22:0)	0.08 (0.03)	0.1 (0.03)	0.17 (0.08)	7.83E-06	1.33E-04	5.48E-02	6.29E-07	1.54E-03
DCER(16:0)	0.1 (0.04)	0.12 (0.06)	0.27 (0.12)	4.89E-07	1.22E-05	7.64E-02	4.09E-08	1.78E-04
CER(16:0)	0.14 (0.05)	0.18 (0.09)	0.38 (0.17)	2.24E-08	1.66E-06	5.06E-02	1.70E-09	5.17E-05
